# Oxidative profiles of LDL and HDL isolated from women with preeclampsia

**DOI:** 10.1186/s12944-017-0480-z

**Published:** 2017-05-16

**Authors:** G. León-Reyes, R. F. Maida-Claros, A. X. Urrutia-Medina, E. Jorge-Galarza, A. M. Guzmán-Grenfell, S. Fuentes-García, R. Medina-Navarro, M. A. Moreno-Eutimio, J. L. Muñoz-Sánchez, J. J. Hicks, Y. D. Torres-Ramos

**Affiliations:** 1 Laboratorio de Regulación Celular, Departamento de Bioquímica, Escuela Nacional de Ciencias Biológicas, Instituto Politécnico Nacional, Ciudad de México, Mexico; 2 Servicio de Neonatología, UCIREN, Instituto Nacional de Perinatología, Secretaría de Salud, Ciudad de México, Mexico; 3 Departamento de Endocrinología, Instituto Nacional de Cardiología, Secretaría de Salud, Ciudad de México, Mexico; 4 Departamento de Inmunobioquímica, Instituto Nacional de Perinatología, Secretaría de Salud, Montes Urales 800, Miguel Hidalgo, Lomas Virreyes, 11000 Ciudad de México, Mexico; 5 Departamento de Metabolismo Experimental, Centro de Investigación Biomédica de Michoacán (CIBIMI-IMSS), Morelia, Michoacán Mexico; 6 Laboratorio de Inmunoquímica, Hospital Juárez de México, Secretaría de Salud, Ciudad de México, Mexico; 7 Comisión Coordinadora de los Institutos Nacionales de Salud y Hospitales de Alta Especialidad, Ciudad de México, Mexico

**Keywords:** Lipoproteins, Oxidative damage, Preeclampsia, Dysfunctional HDL, Endothelial dysfunction

## Abstract

**Background:**

Oxidative stress causes biochemical changes in lipids and proteins; these changes can induce damage to the vascular endothelium and create maternal complications that are characteristic of preeclampsia. In this study, we evaluated the oxidative profile of lipoproteins isolated from women with preeclampsia.

**Methods:**

Thirty women diagnosed with preeclampsia and thirty women without preeclampsia were included in the study. Lipid-damage biomarkers, including conjugated dienes, lipohydroperoxides and malondialdehyde, were measured. The reduction of nitroblue tetrazolium, the formation of dityrosines, and the carbonylation of proteins were assessed as indicators of protein damage. The protective activity of HDL-c was evaluated by the paraoxonase-I activity present on the HDL-c particles. Serum lipid profiles were also quantified in both groups. Data were analysed using Student’s t test and the Pearson correlation coefficient.

**Results:**

Our results demonstrated in PE women evident oxidative changes in the lipids and proteins in HDL-c and LDL-c particles and the activity of the antioxidant enzyme PON-I decreased 59.9%. HDL-c exhibited self-defence, as demonstrated by the negative correlation between paraoxonase-I activity and the formation of lipohydroperoxides in HDL-c (*r* = −0.3755, *p* < 0.005).

**Conclusions:**

LDL-c and HDL-c isolated from women with preeclampsia show oxidative damage to lipids and proteins. We propose an oxidative profile based on the oxidation levels indicated by each of the markers used. We also found that paraoxonase-I is inactivated in the presence of lipohydroperoxides. Antioxidant support might be helpful to reduce oxidative stress in patients with preeclampsia. Further investigations are necessary to define the association between antioxidant activities and preeclampsia.

## Background

Preeclampsia (PE), a syndrome defined by hypertension and proteinuria, is associated with increased maternal mortality and morbidity worldwide [1]. Putative risk factors for PE include nulliparity, family history of PE and chronic hypertension, maternal pre-pregnancy obesity, hypertriglyceridemia, and low dietary and plasma antioxidants. Several of the aforementioned risk factors are also predictive of coronary heart disease in nonpregnant individuals; and the overlap in factors is concentrated among behavioral and metabolic characteristics which are known to be related to lipid metabolism [2].

The cause of PE is largely unknown, but placentation is an important predisposing factor [3]. Whatever the cause of impaired trophoblast invasion, the resulting inadequacy of placental perfusion likely results in oxidative stress by the following mechanisms. The maintenance of the muscular coat of the spiral artery may lead to intermittent placental perfusion because the spiral arteries would retain susceptibility to maternal humoural and neuronal constrictor influences [4]. Together with frequent thrombotic occlusion followed by clot dissolution, this interrupted perfusion may lead to a repeated hypoxia/reoxygenation insult in the affected placenta throughout pregnancy. Hypoxia/reoxygenation is a potent stimulus for the activation of xanthine oxidase and NADPH oxidase, which are important sources of superoxide anion (O_2_^.-^) generation and is abundantly expressed in cytotrophoblasts, syncytiotrophoblasts and villous stromal cells [5]. Placental tissue from women with PE exhibits enhanced xanthine oxidase and NADPH oxidase expression and activity [4].

In PE, this oxidative stress results in the production of the following derivatives of lipid oxidation: conjugated dienes (CDs), lipohydroperoxides (LHPs) and malondialdehyde (MDA). Low density lipoproteins-cholesterol (LDL-c) are particles, which are very susceptible to free radical oxidations. MDA binds to LDL-c, which prevents recognition by its receiver [6]. LDL oxidized (LDLox) may induce functional changes of endothelial cells by stimulating the expression of cell adhesion molecules, which have cytotoxic effects on vascular endothelial cells and are chemoattractant for monocyte and induce macrophage secretion of proinflammatory mediators [7]. High density lipoproteins-cholesterol (HDL-c) have several functions like promoting macrophage cholesterol efflux and reverse cholesterol transport, also HDL-c has been shown to exert direct potentially anti-atherosclerotic, anti-inflammatory and anti-oxidant effects on endothelial cells, such as the direct stimulation of endothelial nitric oxide. PON-I is an HDL-associated esterase that has been shown to protect against lipid peroxide formation in LDL-c and HDL-c [7]. Several authors suggest that HDL-c particles are susceptible to lipid and protein oxidation (the carbonylation of proteins, the formation of dityrosines and reduction of nitroblue tetrazolium), which alters their structure and creates “dysfunctional HDL” particles [8, 9]. HDL-c is a protective agent for vascular function because it contains Apo-AI. The enzyme paraoxonase-I (PON-I) is an esterase that exerts a protective effect against oxidative damage to circulating lipoproteins [10].

The risk of a woman developing PE and the response to oxidative stress depend on several factors, including antioxidant efficacy. Some studies have suggested that antioxidant therapy with vitamin E and vitamin C, which together could inhibit the oxidation of lipids and proteins, could limit the endothelial and utero-placental damage observed in PE [11].

Our objective was to investigate the oxidative changes in lipids and proteins in LDL-c and HDL-c particles isolated from women with PE to expand our knowledge of the biochemical modifications that may explain susceptibility to oxidation.

## Methods

The aim of this study was to evaluate the mechanisms of lipid and protein oxidation in LDL-c and HDL-c lipoproteins isolated from pregnant women with PE. The design of this study was transversal and observational.

### Patients

For this study, 60 women were included and distributed into two groups: 30 women without PE (control group) and 30 women with PE. The inclusion criteria for women in the control group were as follows: normotensive and experienced a normal course of pregnancy according to clinical and ultrasound findings. The inclusion criteria for the PE group were women who matched the diagnostic criteria of the American College of Obstetricians and Gynecologists for PE [1], including blood pressure ≥ 140/90 mmHg and proteinuria ≥300 mg or, in the absence of proteinuria, any of the following conditions: thrombocytopenia, renal insufficiency, impaired liver function, pulmonary oedema or cerebral or visual symptoms. The following exclusion criteria were used for both groups: previous gestational diabetes mellitus, cardiovascular disease, autoimmune diseases, renal or liver diseases, and women who received medication known to interfere with lipids. The rigorous selection, recruitment and collection of biological samples were performed by clinicians of the National Institute of Perinatology (INPer) in Mexico City. All women were informed of the goals of this study and provided their written consent prior to participation in the study. The study was performed according to the principles outlined in the Declaration of Helsinki. The INPer committee for research approved the protocol (212250–3210–21,001-02-14).

### Sample size

We calculated the appropriate sample size using the mean difference formula, and we used the plasma levels of lipohydroperoxides in control women and normotensive women as the reference [12]. The calculated sample size is 17 patients for each group. However, we included 30 patients in the control group and 30 patients with PE.

### Samples

Whole blood (4 mL) was collected from each subject in EDTA tubes (BD Vacutainer, USA) from an antecubital vein before delivery. These samples were centrifuged at 2500 rpm for 15 min to obtain plasma. Plasma was stored at −70 °C until lipid profile analysis and lipoprotein isolation.

### Laboratory procedures

The plasma lipid profile (T-Chol, TAG, HDL-c, Apo-A, and Apo-B), including glutamic oxaloacetic transaminase (GOT), glutamic pyruvic transaminase (GPT), and gamma glutamyl transpeptidase (GGT), were measured in a Hitachi 902 autoanalyser (Boehringer Mannheim) using commercially available kits (Roche Diagnostics, Mannheim Alemania and Wako Chemicals, USA). LDL-c was estimated using the Friedewald formula as modified by DeLong [13].

### Isolation of lipoproteins

Lipoproteins were isolated from plasma at a density of 1.21 g/ml for HDL-c and 1.063 g/mL for LDL-c via sequential preparative ultracentrifugation in a Beckman TL-100 ultracentrifuge at 4 °C. HDL-c was dialyzed against phosphate buffer (pH 7.4) [14].

### Evaluation of lipoperoxidation

CDs were obtained after extraction with chloroform-methanol. The spectrophotometric assay was performed at 234 nm [15]. CDs were quantified using a molar extinction coefficient of 2.7 × 10^4^ M^−1^ cm^−1^, and the results are reported as nmol of CD/mg dry weight. LHP levels were evaluated using the assay conditions described by El-Saadani et al. [16]*.* The calibration curves were obtained using 1 mM t-butylhydroperoxide as standard. The concentration of LHP was calculated using the molar absorptivity of I_3_ measured at 365 nm (є = 2.46 ± 0.25 × 10^4●^M^-1**●**^cm^−1^), and the results are expressed as nmol LHP/mg dry weight. MDA levels were evaluated using 15 mM 1-methyl-2-phenylindole (Sigma-Aldrich, MO, USA) for detection at 586 nm. The values obtained are expressed as nmol of MDA/mg dry weight [17]. The dry weight was determined according to the conditions described by Bernal et al. [18].

### Paraoxonase activity

PON-I activity in HDL-c was evaluated by the hydrolysis of diethyl p-nitrophenyl phosphate (paraoxon), and the absorbance was measured at 405 nm. Enzyme activity was calculated according to the molar extinction coefficient of p-nitrophenol, which is 18,053 (mol/L)^−1^•cm^−1^. Activity is expressed as nmol p-nitrophenol/dl Apo- A/min [19].

### Evaluation of protein damage

Protein damage was evaluated according to the nitroblue tetrazolium (NBT) reduction test, which involved the addition of nitroblue tetrazolium (Sigma-Aldrich) and 2 M glycine (pH 10) according to the method described by Virella et al. [20]*.* The absorbance was read at 530 nm. The results are expressed as nmol formazan/mg protein. Protein carbonylation in the lipoproteins was determined by treatment with 2, 4-dinitrophenylhydrazine (DNPH). DNPH reacts with protein carbonylated (PC) derivatives to form stable hydrazones, which exhibit an absorption peak at 370 nm [21]. A molar extinction coefficient of 21 × 10^3^ M^−1^ cm^−1^ was used to quantify PC content. Values are expressed as nmol PC/mg protein [22]. Dityrosine (DT) levels were determined using a fluorometric assay with 320 nm excitation and 405 nm emission [23]. The results are expressed as nmol DT/mg protein.

### Statistical analysis

The values obtained are presented as the means ± standard deviation. Data were analysed using Student’s t test using Prism 5.0 (Graph Pad, San Diego, CA, USA). The normality of distribution of quantitative variables was analysed using the Kolmogorov Smirnoff test. The Pearson correlation coefficient was used to evaluate correlations. *P* values less than 0.05 were considered significant.

## Results

The control and PE groups included 30 women who met the selection criteria for each group. Women with PE were diagnosed after 20 weeks of gestation, and all presented at least one of the following severe features of PE: blood pressure ≥ 160/110 mmHg, thrombocytopenia, impaired liver function, severe and persistent right upper quadrant or epigastric pain that was unresponsive to medication and not accounted for by alternative diagnoses, progressive renal insufficiency, pulmonary oedema, and new-onset cerebral or visual disturbances [1].

A total of two women (6.6%) in the control group and seven women (23.3%) in the PE group had at least one prior abortion. Women with PE received anti-hypertensive treatment (hydralazine, alpha-methyldopa, nifedipine or labetalol), analgesics (acetaminophen, fentanyl, ketorolac or aspirin) and preventative eclampsia treatments (magnesium sulphate).

Maternal age, pre-pregnancy body mass index (BMI) and gestational age were not significantly different between the groups. All control women delivered by caesarean; 94% of the PE women delivered by caesarean, and 6% delivered vaginally. Compared to the control group, the PE group had higher percentages of elevated systolic/diastolic blood pressure (26.65 and 29.55%, respectively), proteinuria (59.30%), elevated creatinine (an indicator of renal insufficiency, 17.24%), elevated uric acid (34.03%), elevated glucose (31.52%), and impaired liver function indicator enzymes GOT (33.40%), GPT (81.04%) and GGT (103.17%). The PE group exhibited LDL-c levels that were 51.56% higher than those in the control group. T-Chol, TAG, HDL-c, Apo-A and Apo-B levels did not differ significantly between groups (Table 1).

**Table 1:** Demographic and clinical data

Characteristics	Control	Preeclampsia	Statistical significance
Number	30	30	---
Age (years)	30.91 ± 5.94	30.39 ± 6.60	*p* = 0.7796
Pre-pregnancy BMI (kg/m^2^)	26.66 ± 5.16	27.42 ± 3.62	*p* = 0.5946
Gestational age (weeks)	38.3 ± 1.10	37.5 ± 2.18	*p* = 0.1315
Delivery: vaginal/caesarean (%)	0/100	6/94	---
Systolic blood pressure (mm Hg)	*114.8 ± 9.76*	*145.4 ± 16.60*	*p* *<* *0.0001*
Diastolic blood pressure (mm Hg)	*70.81 ± 7.25*	*91.74 ± 10.87*	*p <* *0.0001*
Proteinuria (mg/24 h)	*263.2 ± 73.96*	*419.3 ± 169.0*	*p=0.0388*
Creatinine (mg/dl)	*0.58 ± 0.14*	*0.68 ± 0.11*	*p=0.0055*
Uric Acid (mg/dl)	*4.26 ± 1.38*	*5.71 ± 1.41*	*p<0.001*
Glucose (mg/dl)	*72.27 ± 19.99*	*95.05 ± 29.86*	*p=0.0003*
GOT (U/L)	*22.60 ± 12.17*	*30.15 ± 18.15*	*p=0.0341*
GPT (U/L)	*12.24 ± 5.10*	*22.16 ± 11.78*	*p=0.0441*
GGT (U/L)	*11.64 ± 7.3*	*23.65 ± 12.5*	*p=0.0345*
T-Chol (mg/dl)	192.9 ± 62.43	213.5 ± 58.33	*p* = 0.1513
TAG (mg/dl)	236.8 ± 76.35	280.5 ± 115.1	*p* = 0.0613
HDL-c (mg/dl)	51.31 ± 18.16	49.07 ± 16.82	*p* = 0.5895
LDL-c (mg/dl)	*108.6 ± 38.66*	*164.6 ± 83.02*	*p=0.0004*
Apo-A (mg/dl)	178.5 ± 49.51	186.1 ± 46.53	*p* = 0.5076
Apo-B (mg/dl)	111.2 ± 37.32	125.8 ± 43.94	*p* = 0.1346

Statistical values were obtained after a comparison between preeclamptic women (*n* = 30) and the control group (*n* = 30). Values are presented as the means ± SD. The values in italic letter represent statistical significance (*p* < 0.05)

**Fig. 1:**
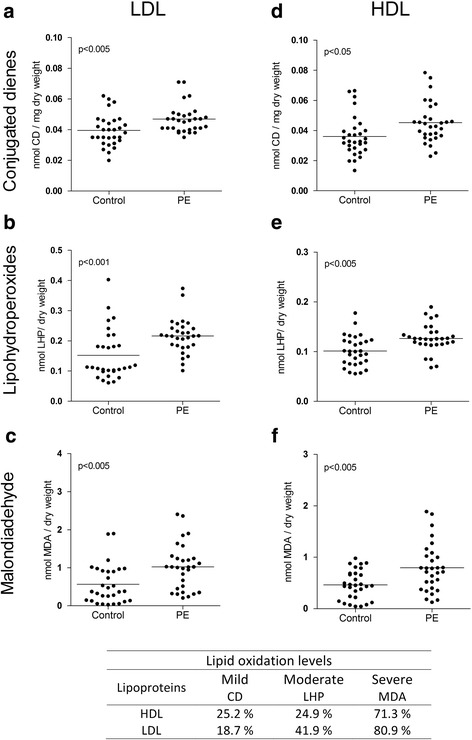
Biomarkers of oxidative damage to lipids in LDL-c and HDL-c particles. Biomarkers of damage to lipids in LDL-c and HDL-c. Conjugated dienes (**a** and **d**), lipohydroperoxides (**b** and **e**) and malondialdehyde (**c** and **f**). Statistical values and oxidation levels were obtained after comparison between preeclamptic women (*n* = 30) and controls (*n* = 30). The data are shown in scatter plots, and the mean population is indicated. Statistical significance (*p* < 0.05)

Figure 1 shows the levels of the three biomarkers of lipid damage in LDL-c (Fig. 1a, b and c) and HDL-c (Fig. 1d, e,  and f). Increased percentages of CDs (18.7 and 25.2%), LHPs (41.9 and 24.9%), and MDA (80.9 and 71.3%) in LDL-c and HDL-c, respectively, were seen in PE women compared to the percentages in the control group. Obviously greater damage was also observed in LDL-c compared to HDL-C. These results were grouped by severity of lipid oxidation (Fig. 1) corresponding to the degree of oxidation as follows: CDs, mild oxidative damage; LHP, moderate oxidative damage; and MDA, severe oxidative damage.

**Fig. 2:**
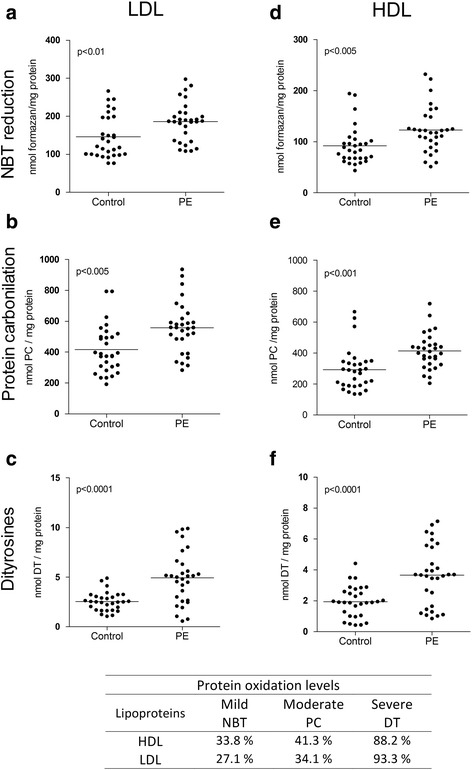
Biomarkers of oxidative damage to proteins in LDL-c and HDL-c particles. Biomarkers of damage to proteins in LDL-c and HDL-c. NBT reduction (**a** and **d**), protein carbonilation (**b** and **e**) and dityrosines (**c** and **f**). Statistical values and oxidation levels were obtained after comparison between preeclamptic women (*n* = 30) and controls (*n* = 30). The data are shown in scatter plots, and the mean population is indicated. Statistical significance (*p* < 0.05)

Figure 2 shows the levels of protein damage biomarkers in LDL-c (Fig. 2a, b, and c) and HDL-c (Fig. 2d, e, and f). PE women exhibited increased levels of NBT-reduction (27.1 and 33.8%), PC (34.1 and 41.3%), and DT (93.3 and 88.2%) in LDL-c and HDL-c, respectively, compared to the control group. These results were grouped into levels of protein oxidation (Fig. 2) as follows: NBT reduction, mild; PC, moderate; and DT, severe oxidation.

**Fig. 3:**
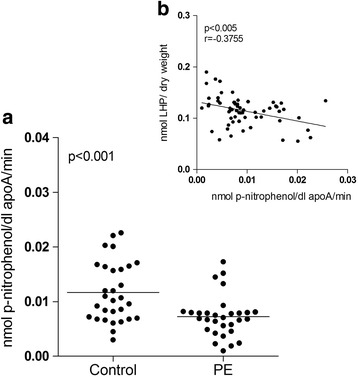
Paraoxonase-I activity. PON-I activity in preeclamptic women (*n* = 30) compared with the control group (*n* = 30). The data are shown in scatter plots, and the mean population is indicated (**a**). Relationship between PON-I activity and lipohydroperoxide levels in HDL-c (**b**)

The activity of the antioxidant enzyme PON-I decreased 59.9% in PE women compared to the control group (Fig. 3a). A significant correlation was observed between the decrease in PON–I activity and high LHP levels in HDL-c, and the correlation coefficient was −0.3755 (*p* < 0.005) (Fig. 3b).

## Discussion

PE shares conventional risk factors with cardiovascular disease, such as obesity, dyslipidaemia, hypertension, and insulin resistance [24]. The common basis for these disorders is the presence of endothelial dysfunction. One pathway of endothelial dysfunction is related to alterations in the plasma levels of lipids and apolipoproteins as sources of lipoperoxidation and oxidative stress [25].

Several studies have demonstrated the susceptibility of LDL-c and HDL-c to oxidation in women with PE. These studies conclude that LDL-c and HDL-c particles are more susceptible to oxidative modification and that the plasma concentration of LDL-c particles but not HDL-c particles is increased in PE [10], results that are consistent with the current investigation (Table 1).

**Fig. 4.**
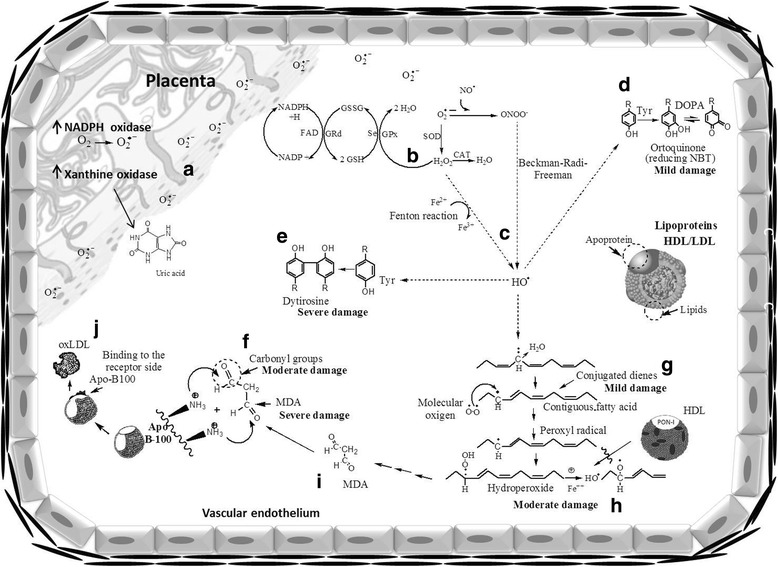
Mechanisms of oxidative damage in LDL-c and HDL-c. The main source of reactive species in PE is the placenta via the NADPH oxidase and xanthine oxidase production of O_2_^.-^ (**a**), which is dismutated by SOD to produce H_2_O_2_, which can be removed by CAT and GPx. GPx uses GSH as a reducing agent, and GSH is regenerated by GRd (**b**). When this antioxidant system is disrupted, excess O_2_^.-^ can react in two ways. It can interact with NO^•^ to produce ONOO^−^ via the Beckman-Radi-Freeman reaction, and ONOO^−^ can become protonated and rearrange to produce HO^•^. Alternatively, SOD produces H_2_O_2_, which can interact with transition metals (Fenton reaction) to generate HO^•^ (**c**). HO^•^ can modify proteins and lipids from lipoproteins. Damage to proteins may occur via three oxidation mechanisms: 1) the modification of tyrosine residues to generate catechols (DOPA) when oxidized that then form orthoquinones that can reduce the nitroblue tetrazolium compound (NBT) (mild damage) (**d**), 2) the production of dimeric tyrosines (dityrosines) (severe damage) (**e**) or 3) the formation of carbonyl groups generated by direct free radical attack, interactions with transition metals, glycation and adduct formation between protein and lipoperoxidation products (**f**). HO^•^ may also oxidize lipids, which undergo molecular rearrangements to form conjugated dienes (mild damage) (**g**), lipohydroperoxides (moderate damage) (which may be hydrolysed by PON-I present on HDL) (**h**), and MDA (severe damage) (**i**). MDA can form adducts with apo B-100 of LDL and generate oxLDL, which is toxic to the vascular endothelium (**j**)

Notably, the main source of reactive oxygen species in PE is the placenta due to the activity of NADPH oxidase and xanthine oxidase [5], which generate O_2_^.-^ (Fig. 4a). This radical is dismutated by superoxide dismutase to produce H_2_O_2_, which is removed by catalase and glutathione peroxidase. Glutathione peroxidase uses reduced glutathione (GSH), which is regenerated by glutathione reductase (GRd) using NADPH as a reducing agent (Fig. 4b) [6]. When this antioxidant system is disrupted, excess O_2_^.-^ can react in two ways to form the hydroxyl radical (HO^•^): 1) O_2_^.-^ can react with NO^•^ to produce ONOO^−^ via the Beckman-Radi-Freeman reaction [6], or 2) H_2_O_2_ can interact with transition metals (iron or copper) in what is known as the Fenton reaction [6] (Fig. 4c).

HO^•^ oxidizes polyunsaturated lipids, which undergo molecular rearrangements and form CDs (Fig. 4g), LHP (Fig. 4h), and MDA (Fig. 4i). The measurement of lipoperoxidation products in HDL-c and LDL-c particles of women with PE in our study revealed 25.2 and 18.7% increases, respectively, in CDs; 24.9 and 41.9% increases, respectively, in LHP; and 71.3 and 80.9% increases, respectively, in MDA (Fig. 1). The products of lipoperoxidation were associated with an increase in the percentage of oxidative damage in maternal lipoproteins. Therefore, we classified oxidative damage to lipids into three stages: CDs were associated with mild damage, LHP was associated with moderate damage, and MDA was associated with severe damage. In the literature, these oxidation products are classified according to the steps of the process; CDs indicate initiation, LHP indicates propagation and MDA indicates termination [6].

With respect to the oxidation of proteins, it is known that the damage is caused by HO^•^ via the following three mechanisms: 1) the modification of tyrosine residues, which generate catechols (DOPA) and then form orthoquinones when oxidized and reduce NBT in the presence of glycine (Fig. 4d) to create formazan [20]; 2) the production of tyrosine dimers (dityrosines) (Fig. 4e) [23]; and 3) the PC, which is caused by the direct attack of free radicals, by interaction with transition metals, by glycation or by the formation of adducts with MDA (final product of lipoperoxidation) [6] (Fig. 4f). Our results support the classification of the intensity of protein damage in HDL-c and LDL-c of women with PE. The production of formazan is associated with mild damage and exhibited an increase of 33.8% in HDL-c and 27.1% in LDL-c. The PC is associated with moderate damage and increased 41.3% in HDL-c and 34.1% in LDL-c. The formation of dityrosines is associated with severe damage and increased 88.2% in HDL-c and 93.3% in LDL-c.

The susceptibility of lipoproteins to oxidation processes may be interpreted as a decrease in antioxidant mechanisms [26]. Previous studies reported that women with PE exhibit a decrease in the activity of serum PON-I, which represents antioxidant activity because of its ability to remove LHPs from lipoproteins, especially LDL-c, and to modulate the levels of oxidative stress [12]. Our results revealed a 59.9% decrease in PON-I activity in the HDL-c particles of women with PE (Fig. 3a). This result may explain why the exposure of HDL-c particles to oxidation in PE women affects PON-I activity and generates a “dysfunctional HDL”, as noted previously [8, 9]. Another cause of the decrease in PON-I activity in women with PE is the liver damage which are associated with the elevation of TGO and TGP enzymes and may affect PON-I synthesis. Modified HDL-c is unable to defend LDL-c from oxidation under these circumstances. The presence of oxidized lipids in HDL-c has been proposed to play a role in the altered antioxidant properties of HDL-c [2]. However, this damaged HDL-c may retain some self-defence because a negative correlation (*p* < 0.005, *r* = −0.3755) was observed between PON-I activity and LHP levels in HDL-c. Plasma HDL-c levels were significantly decreased, and maternal vascular function was reduced. Wire myography studies demonstrated an association between plasma Apo-AI content, which is the major protein constituent of HDL-c, and blood vessel relaxation [24]. These observations suggest that HDL-c concentration and function increase in pregnancy to protect the maternal vascular endothelium and that this increase fails to occur in PE [24].

Although this study reached its aims, a limitation was the control of variables in the patients’ lifestyles, such as diet, physical activity, and traffic-generated air pollution, variables that were not analysed in this study and have been reported to influence the risk of PE, reproductive outcomes, and preterm birth. This limitation should be considered in the design of future studies.

## Conclusions

Our results show that HDL-c and LDL-c isolated from women with PE present oxidative damage to lipids and proteins. These results allowed us to propose an oxidative profile based on the oxidation levels indicated by each of the markers used. Increased oxidative stress in PE may lead to low PON-I activity; we found that PON-I is inactivated in the presence of lipohydroperoxides. Additionally, there was a negative correlation between PON-I and LHP in HDL-c in our study. Antioxidant support might be helpful to reduce oxidative stress in patients with PE. Further investigations are necessary to define the association between antioxidant activities and PE. It is important to implement diets rich in antioxidants (fruits, grains, and vegetables) to improve the quality of life of pregnant women, and recommends its consumption during the Inter-Pregnancy intervals as well, and finally consider the supervised antioxidant supplementation.

## Abbreviations

Apo-A: Apoprotein A
Apo-B: Apoprotein B
BMI: Body mass index
CDs: Conjugated dienes
DNPH: 2,4-Dinitrophenyl hydrazine
DT: Dityrosine
GGT: Gamma glutamyl transpeptidase
GOT: Glutamic oxaloacetic transaminase
GPT: Glutamic pyruvic transaminase
HDL: High Density Lipoprotein
LDL: Low Density Lipoprotein
LHP: Lipohydroperoxide
MDA: Malondialdehyde
NBT: Nitroblue tetrazolium
PC: Protein carbonylated
PE: Preeclampsia
PON-I: Paraoxonase-I
TAG: Triglycerides
T-Chol: Total cholesterol
